# Effects of Zirconium Doping Into a Monoclinic Scheelite BiVO_4_ Crystal on Its Structural, Photocatalytic, and Photoelectrochemical Properties

**DOI:** 10.3389/fchem.2018.00266

**Published:** 2018-07-02

**Authors:** Shigeru Ikeda, Takato Kawaguchi, Yui Higuchi, Naoto Kawasaki, Takashi Harada, Mikas Remeika, Muhammad M. Islam, Takeaki Sakurai

**Affiliations:** ^1^Department of Chemistry, Konan University, Hyōgo, Japan; ^2^Research Center for Solar Energy Chemistry, Osaka University, Osaka, Japan; ^3^Institute of Applied Physics, University of Tsukuba, Ibaraki, Japan

**Keywords:** bismuth vanadate, Zr-doping, water oxidation, crystalline structure, photocatalysis

## Abstract

Effects of zirconium (Zr) doping into BiVO_4_ powder on its structural properties and photocatalytic activity for O_2_ evolution were examined. The formation of BiVO_4_ powder crystallized in a monoclinic scheelite structure (*ms*-BiVO_4_) was achieved when the sample was doped with a relatively small amount of Zr. The photocatalytic activity of Zr-doped *ms*-BiVO_4_ powder was much higher than that of non-doped *ms*-BiVO_4_. However, further doping caused a reduction of photocatalytic activity for O_2_ evolution due to the occurrence of structural alterations into tetragonal scheelite and tetragonal zircon structures. Similar effects of Zr doping were also observed for the photoelectrochemical (PEC) system based on BiVO_4_ thin films doped with various amounts of Zr. Thus, Zr doping was confirmed to be effective for improvements of photocatalytic and PEC functions of BiVO_4_ for water oxidation.

## Introduction

Bismuth vanadate (BiVO_4_), an *n*-type metal oxide semiconductor, has been widely studied as a potential photocatalyst and/or photoanode for oxygen (O_2_) production through water-splitting using sunlight due to its suitable band-gap energy (E_g_), high optical absorption, reasonable band-edge alignment for O_2_/H_2_O redox potential, nontoxicity, and abundance of constituent elements (Kudo et al., 1998, 1999; Park et al., 2013; Suarez et al., 2015). Although BiVO_4_ cannot produce hydrogen (H_2_) from water because of its insufficient electron energy level of the conduction band minimum (CBM) (Kudo et al., 1999; Kato et al., 2004; Walsh et al., 2009), the compound has been used for a two-step photoexcitation system, called a Z-scheme water splitting, that is analogous to natural photosynthesis (Kato et al., 2004; Sasaki et al., 2009; Iwase et al., 2011; Martin et al., 2014; Jiang et al., 2015; Baek et al., 2017).

BiVO_4_ exists in three crystalline phases, namely, monoclinic scheelite (*ms*-BiVO_4_), tetragonal scheelite (*ts*-BiVO_4_), and tetragonal zircon (*tz*-BiVO_4_) structures (Lim et al., 1995). For water oxidation into O_2_, the first two scheelite structures are known to be active; enhanced photoactivity of scheelite BiVO_4_ compounds is mainly due to enhanced photon absorption properties derived from their relatively narrow band-gap energies (2.4 eV) compared to that of *tz*-BiVO_4_ (2.9 eV) (Kudo et al., 1999). Among the scheelite compounds, moreover, the *ms*-BiVO_4_ phase shows much higher activity for O_2_ production from water than does the *ts*-BiVO_4_ phase. The difference is likely to be due to induction of local polarization owing to an appreciable lattice distortion of the Bi-O bond in *ms*-BiVO_4_ compared to that in *ts*-BiVO_4_, leading to the enhancement of efficient electron-hole separation (Tokunaga et al., 2001).

BiVO_4_ possess two doping sites, Bi and V. Doping with molybdenum (Mo) or tungsten (W) at the V site has been shown to be effective for enhancing activity for water oxidation (Park et al., 2011; Ye et al., 2011; Berglund et al., 2012). Since a hexavalent form is the most stable phase for Mo and W, doping with these elements at the pentavalent V site should introduce excess electrons. Hence, increasing *n*-type conductivity would be effective for enhancement of the activity, though details in correlations between the enhancement of photocatalytic activity and carrier dynamics of those doped compounds have been less discussed yet (Grigioni et al., 2015). In addition, according to the study of density functional theory (DFT) (Yin et al., 2011), group IVB element, i.e., Zr and Hf, can substitute the Bi site in BiVO_4_ with low formation energy. Since replacement of trivalent Bi with tetravalent Zr or Hf induces enhancement of *n*-type conductivity, doping of these elements is expected to have effects that are similar to those of doping with Mo or W. In the present study, therefore, BiVO_4_ powders and films doped with Zr were prepared. Photocatalytic and PEC properties for water oxidation were investigated in relation to their structural characteristics.

## Experiment

### Preparation of powder photocatalysts

BiVO_4_ powder was prepared in an aqueous nitric acid solution containing Bi^3+^ ions and V_2_O_5_ powder, as reported in the literature (Ng et al., 2010; Iwase et al., 2011). A 4.9-g portion of Bi(NO_3_)_3_·5H_2_O (Wako) was dissolved in 50 mL of 0.75 M nitric acid (Bi-soln). After addition of 0.92 g of V_2_O_5_ (Wako) to Bi-soln, the orange-colored suspension was stirred at room temperature for 48 h. A yellow powder suspension thus-obtained was filtered to collect the powder part. The powder was washed several times with water and then dried at 70°C. The thus-obtained non-doped BiVO_4_ powder was labeled BVO. For doping with Zr, appropriate amounts of ZrO(NO_3_)_2_·H_2_O (Wako) were dissolved in Bi-soln, and the reaction was performed by the same procedure as that for the preparation of non-doped BiVO_4_. The amounts of doped Zr were varied from 0.1 to 3.0% by changing the amount of ZrO(NO_3_)_2_·H_2_O added to Bi-soln. Samples as-obtained were designated Zr(x)BVO, where x denotes the molar content of doped Zr. For example, Zr(0.5)BVO represents 0.5 mol% of Zr-doped BiVO_4_. For comparison, BiVO_4_ powder doped with 0.5% of Mo [labeled M(0.5)BVO] was also prepared through the same process using a Bi-soln solution containing 8.9 mg of (NH_4_)_6_[Mo_7_O_24_]·4H_2_O (Wako).

### Preparation of thin-film electrodes

BiVO_4_ thin films were prepared on fluorine-doped tin-oxide-coated glass slides (FTO/glass, Aldrich) by spin coating in accordance with the literature procedure (Fuku and Sayama, 2016) with same modifications. Typically, a mixed solution (Bi: V = 1: 1) of bismuth oxide of SYM-BIO5 enhanced metal organic decomposition material (EMOD, Symetrix) and vanadium oxide of V-02 DIP COAT-PRECURSORS (High Pure Chemicals) dissolved in butyl acetate (Wako) containing ethylcellulose (Wako) as a thickener and an aggregation inhibition agent was coated on FTO/glass by spin coating (500 rpm, 15 s). Then the film was calcined at 550°C for 30 min. For Zr doping, appropriate amounts of zirconium oxide of SYM-ZRO5 EMOD (Symetrix) were added to the mixed solution; the same procedure as that for fabrication of the non-doped BiVO_4_ thin film was used. In the same manner as that for the above powder system, thus-obtained non-doped BiVO_4_ and Zr-doped BiVO_4_ powders were labeled BVO_*f*_ and Zr(x)BVO_*f*_ (x: molar amount of doped Zr), respectively.

### Characterization of powder and thin-film samples

Crystalline structures of samples were analyzed by X-ray diffraction (XRD) using a Rigaku Mini Flex X-ray diffractometer (Cukaα, Ni filter). Morphologies of powder samples were examined by using a Hitachi S-5000 FEG field emission scanning electron microscope (SEM) at an acceleration voltage of 20 kV. Raman spectroscopy measurements using a JASCO NRC 3100 Laser Raman Spectrophotometer (excitation laser having a wavelength of 532 nm) were performed for structural analyses of thin-film samples.

### Photocatalytic and PEC reactions

For photocatalytic O_2_ evolution, 50 mg of a powder sample and 5 cm^3^ of 0.05 M aqueous AgNO_3_ (Wako) solution were added to a borosilicate-glass test tube. After replacement of air to Ar in the test tube, the suspension was photoirradiated by a Parkin Elmer CERMAX LX-300BUV Xe lamp. The amount of evolved O_2_ was analyzed every 0.5 h by using a Shimadzu GC-8A gas chromatograph equipped with an MS-5A column (GL sciences) and a TCD detector. PEC water oxidation was performed by using a three-electrode system consisting of the thin-film sample as a working electrode, a Pt wire as a counter electrode, and Ag/AgCl as a reference electrode. These electrodes were immersed in a phosphate buffer with pH 6.86 (Wako). The above-mentioned Xe lamp was used as a light source.

## Results and discussion

### Structural and photocatalytic properties of Zr-doped BiVO_4_ powders

**Figure 1:**
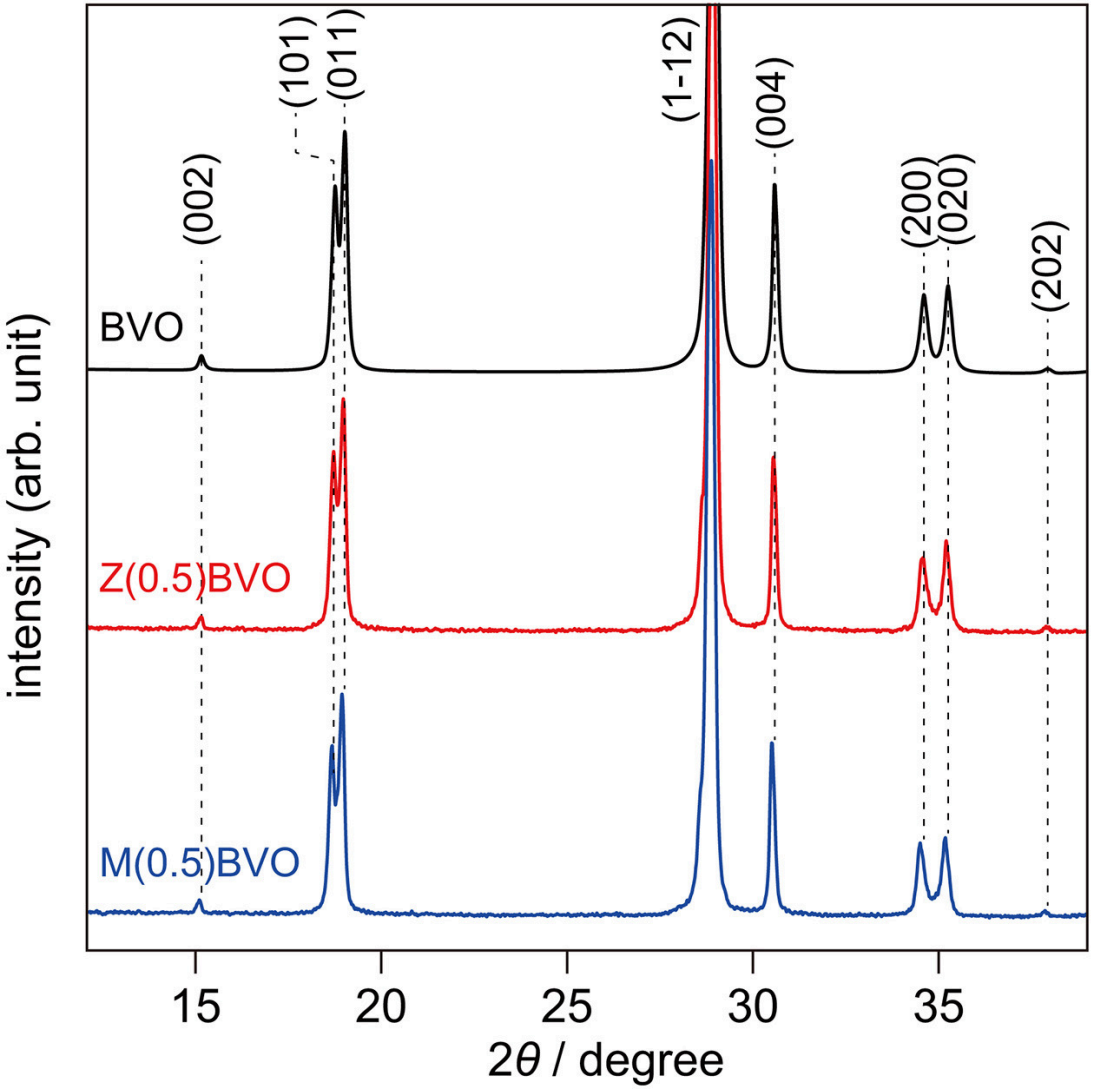
XRD patterns of BVO, Zr(0.5)BVO, and Mo(0.5)BVO powders.

Figure 1 shows XRD patterns of BVO, Zr(0.5)BVO, and Mo(0.5)BVO powders. For all of the samples, observed diffractions were assignable to scheelite BiVO_4_; there was no diffraction derived from any other impurities. Moreover, observation of two reflections at 2θ of *ca*. 19°, indexed to the (101) and (011) reflections, indicates that crystalline structures of these powders are classified to the monoclinic crystal system (JCPDS 75-1866). Similar behavior was also observed for the peaks at 2θ of *ca*. 35° which are indexed to the (200) and (020) reflections of *ms*-BiVO_4_ (JCPDS 75-1866). It should be noted that the XRD pattern of M(0.5)BVO showed slight shifts of all of the peaks toward low 2θ angle regions. Since the ionic radius of Mo^6+^ (0.062 nm) is larger than that of V^5+^ (0.052 nm) (Yin et al., 2011), the observed shifts indicate incorporation of Mo at the V site in the *ms*-BiVO_4_ crystalline lattice. On the other hand, there was no appreciable peak shift observed for the Zr(0.5)BVO sample: occurrence of peak shifts toward high 2θ angle regions was expected when the doped Zr^4+^ (0.079 nm) was assumed to occupy the Bi^3+^ (0.111 nm) site (Grigioni et al., 2015). However, there was no appreciable shift observed. Further studies for identification of the doping site(s) for the present Zr dopant are needed.

**Figure 2:**
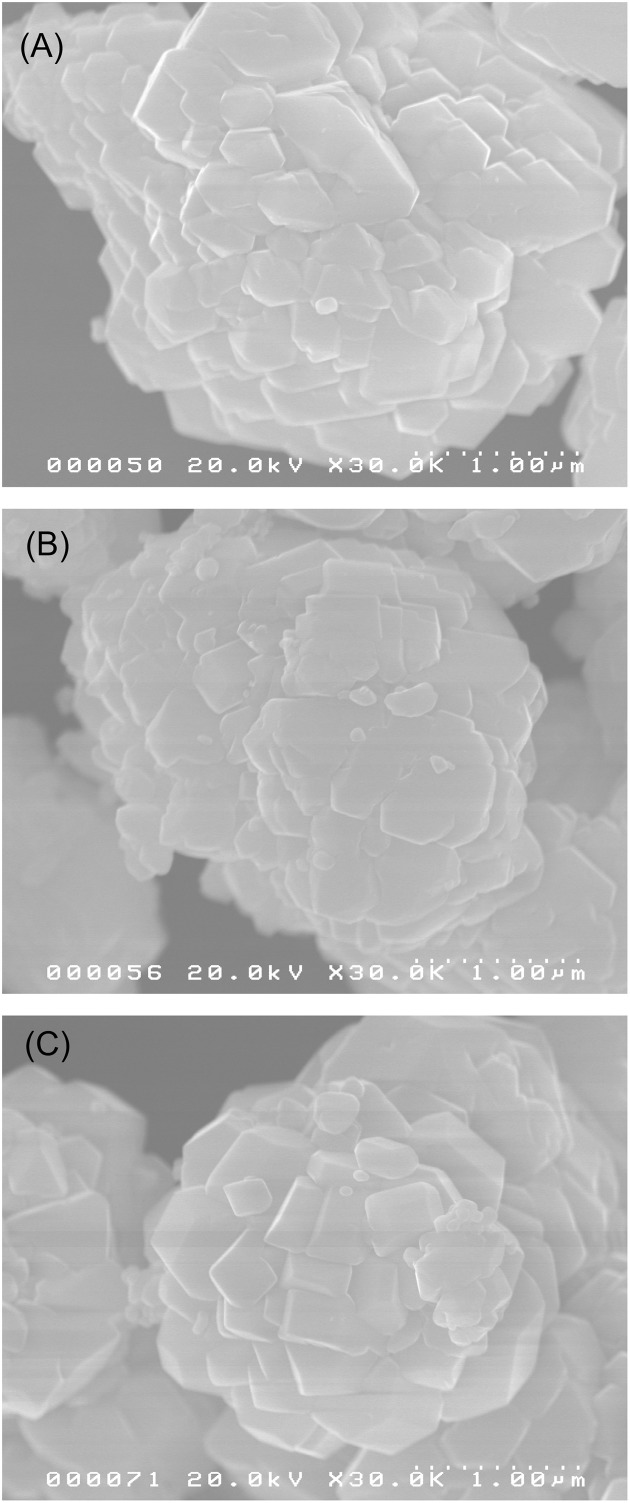
SEM images of **(A)** BVO, **(B)** Zr(0.5)BVO, and **(C)** Mo(0.5)BVO powders.

Figure 2 shows typical SEM images of BVO, Zr(0.5)BVO, and Mo(0.5)BVO powders. The BVO sample consisted of aggregates of angular shaped crystals with submicron sizes. Similar morphologies were also observed for Zr(0.5)BVO and Mo(0.5)BVO powders. As for the results of above XRD analyses are concerned, there are almost no structural and morphological differences between these samples.

**Figure 3:**
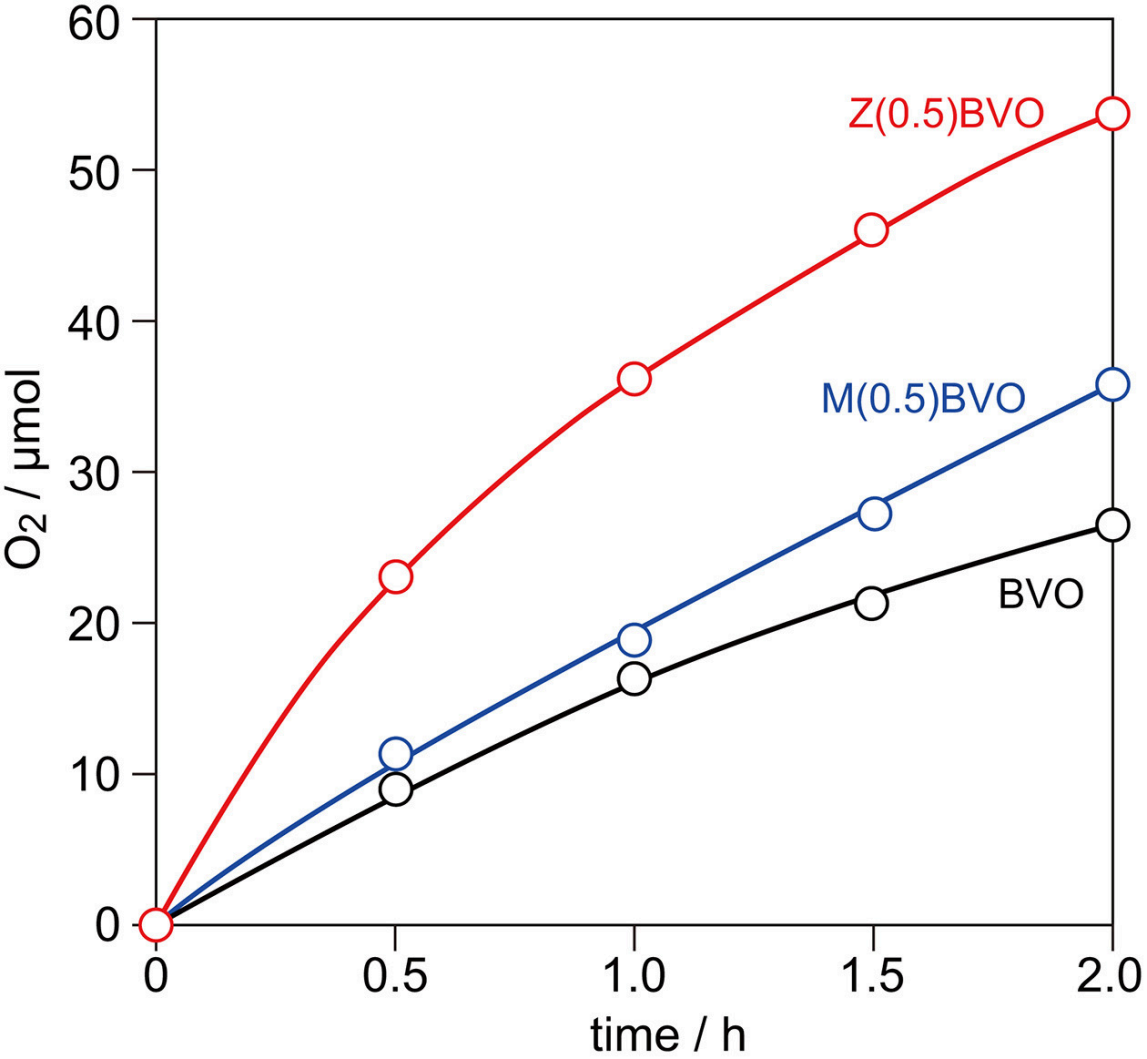
Time course curves of O_2_ evolution from 0.05 M aqueous AgNO_3_ solution over BVO, Zr(0.5)BVO, and Mo(0.5)BVO powders under photoirradiation with a 300 W Xe lamp in Ar.

For the evaluation of photocatalytic activity for water oxidation, BVO, Zr(0.5)BVO, and Mo(0.5)BVO powders were photoirradiated with a Xe lamp (E_g_ > 2.4 eV) in an aqueous Ag(NO_3_)_3_ solution under Ar. Figure 3 shows typical time course curves of O_2_ evolution over these powders. As expected from the literature, all of the samples having a monoclinic scheelite structure exhibited appreciable activity for this reaction. Samples, especially the Zr(0.5)BVO sample, showed higher activity than that of the BVO sample. Although the effectiveness of Mo doping is well known in the literature (Park et al., 2011; Ye et al., 2011; Berglund et al., 2012; Wang et al., 2016) and the effectiveness of Zr doping for methylene blue photodegradation have been investigated (Karunakaran et al., 2014), the results of the present study are the first demonstration of enhancement of photocatalytic activity for water oxidation induced by Zr doing into the *ms*-BiVO_4_ lattice.

**Figure 4:**
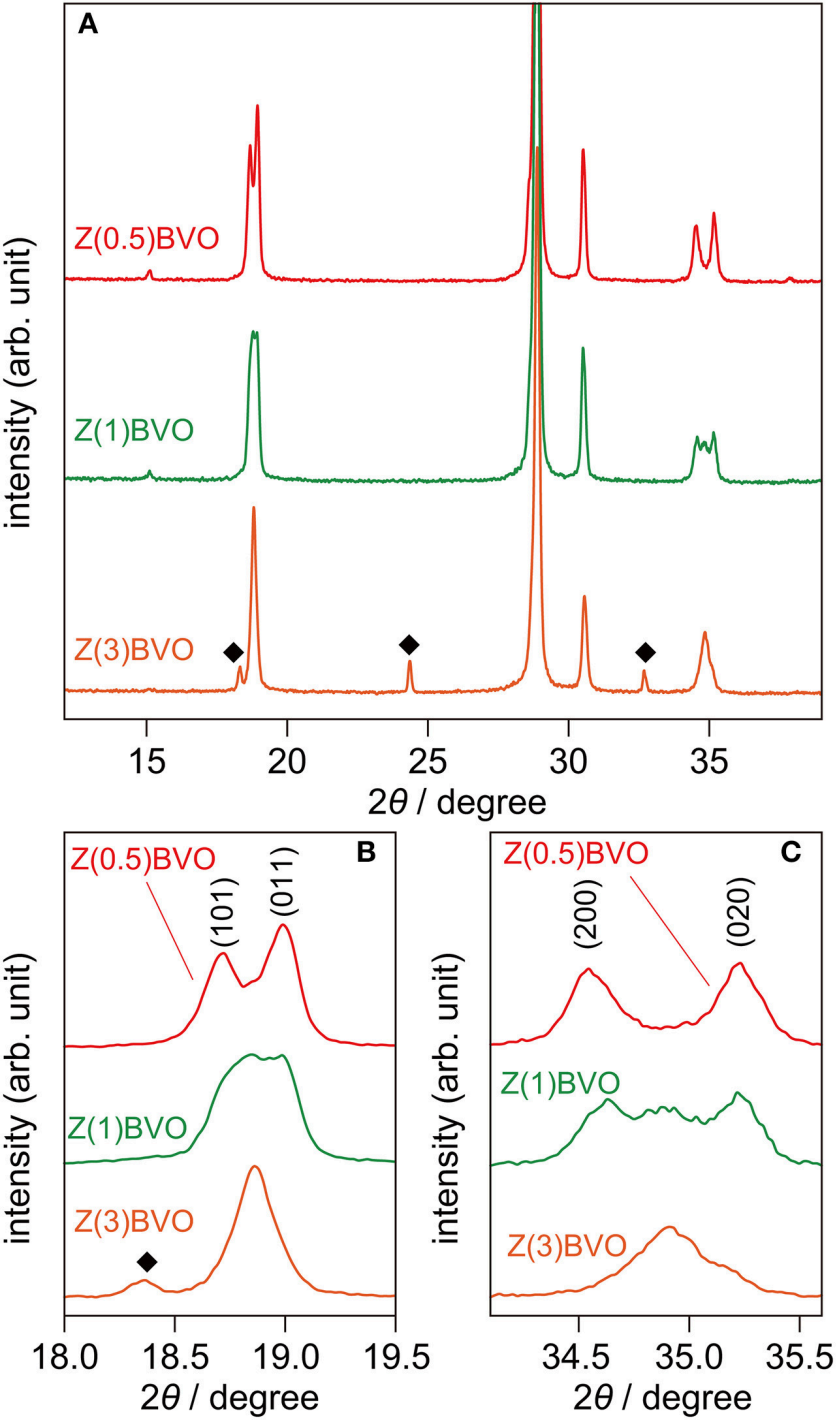
**(A)** XRD patterns of Zr(0.5)BVO, Zr(1.0)BVO, and Mo(3.0)BVO powders. **(B,C)** Magnified views at 2θ ranges of 18.0°-19.5° and 34.1°-35.6°, respectively.

**Figure 5:**
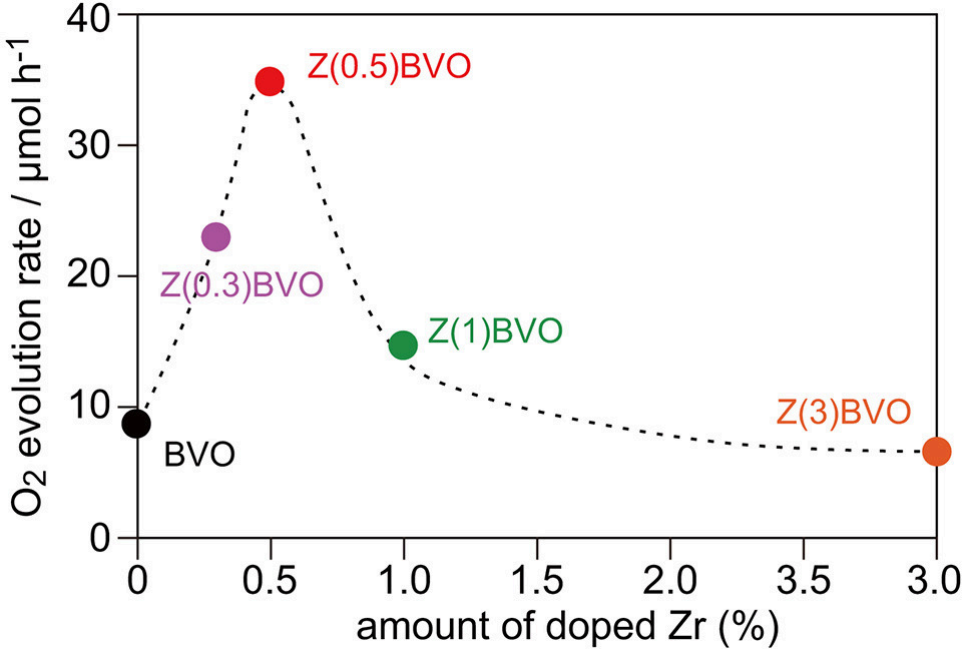
Dependence of rate of O_2_ evolution on amount of doped Zr. Photocatalytic reactions were performed in 0.05 M aqueous AgNO_3_ solutions under photoirradiation with a 300 W Xe lamp in Ar.

Next, the effects of different amounts of Zr doping in the BiVO_4_ lattice on structural and photocatalytic properties were examined. Figure 4 shows XRD patterns of powder samples with various amounts of doped Zr. It is clear from the figure that appreciable structural alterations occurred upon increment of the Zr content. In the XRD pattern of the Zr(1.0)BVO sample, appreciable changes in the diffraction pattern were observed at 2θ of *ca*. 19° and *ca*. 35°: additional reflections appeared between the (101) and (011) reflections and also between the (200) and (002) reflections (Figures 4A,B). (Park et al., 2011; Berglund et al., 2012) These changes were pronounced for the Zr(3.0)BVO sample: these reflections merge into single peaks in the XRD pattern of the sample, indicating structural transition into tetragonal scheelite (*ts*-BiVO_4_) (JCPDS 14-0133). It should be noted that several peaks other than the scheelite form, which are assignable to diffractions of the tetragonal zircon form of BiVO_4_ (*tz*-BiVO_4_), were also observed in the XRD pattern of the Mo(3.0)BVO sample. As can be expected from the literature, these structural transitions were detrimental to photocatalytic activity. Indeed, the rate of O_2_ evolution, defined as the amount of O_2_ evolved in the first hour of photoirradiation, reached a maximum for the Zr(0.5)BVO sample and tended to decrease with an increase in the amount of doped Zr as shown in Figure 5. It should be noted that the highest activity was also obtained over the Mo(0.5)BVO sample for the Mo-doped system, and further doping was detrimental to photocatalytic activity because of the occurrence of structural transitions similar to those for the Zr-doped system (data not shown).

### PEC O_2_ evolution over Zr-doped BiVO_4_

**Figure 6:**
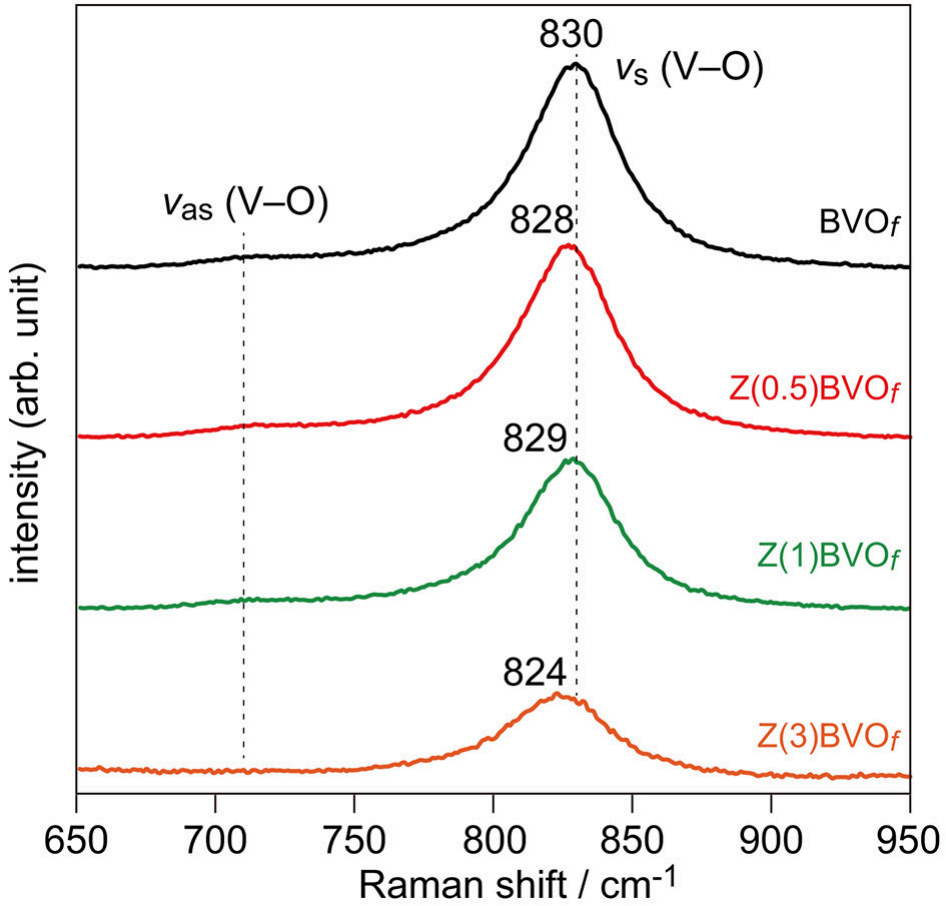
Raman spectra of BVO_*f*_, Zr(0.5)BVO_*f*_, Zr(1.0)BVO_*f*_, and Zr(3.0)BVO_*f*_ films deposited on FTO/glass substrates.

In order to examine the impact of Zr doping on the PEC property for O_2_ evolution over the BiVO_4_ photoanode, Zr-doped BiVO_4_ thin films were fabricated on FTO/glass substrates. Figure 6 shows Raman spectra of thus-obtained BVO_*f*_, Zr(0.5)BVO_*f*_, Zr(1.0)BVO_*f*_, and Zr(3.0)BVO_*f*_ films. The dominant Raman bands observed in all of the samples at *ca*. 830 cm^−1^ were assigned to the symmetric V-O stretching mode (ν_s_(V-O)); weak shoulders at *ca*. 710 cm^−1^ were assigned to the asymmetric V-O stretching mode (ν_as_(V-O)). Both of the Raman signals are typically observed in *ms*-BiVO_4_. It should be noted that slight red shifts of ν_s_(V-O) bands were observed for Zr-doped samples with relatively low Zr contents (Zr(0.5)BVO_*f*_ and Zr(1.0)BVO_*f*_). Since similar red shifts were reported in the literature for the Mo-doped *ms*-BiVO_4_ system (Merupo et al., 2015), these shifts would be due to incorporation of Zr into the crystalline lattice of *ms*-BiVO_4_. The red shifts were pronounced with an increase in the amount of doped Zr (*i.e*., Zr(3.0)BVO_*f*_). Appreciable broadening was also observed for the ν_s_(V-O) Raman band of the sample. The fact that *ts*-BiVO_4_ exhibits the ν_s_(V-O) band at 823 cm^−1^ (Nikam and Joshi, 2016) suggests incorporation of the *ts*-BiVO_4_ phase in the sample, as expected from the above-described results for the powder system.

**Figure 7:**
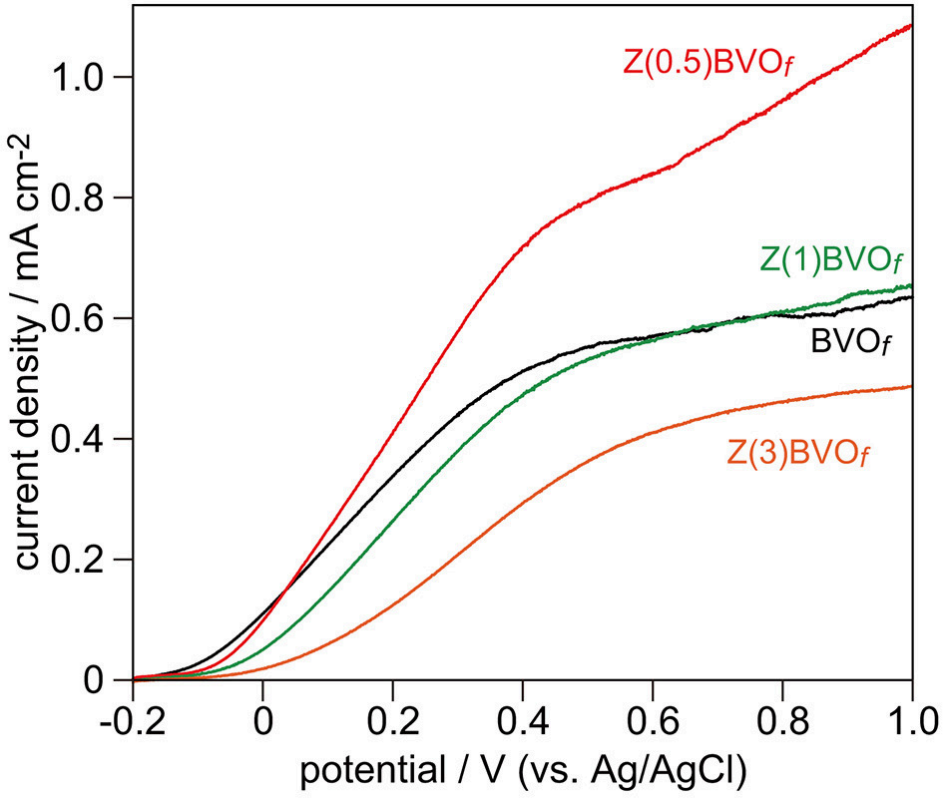
Linear sweep voltammogram (LSV) scans of BVO_*f*_, Zr(0.5)BVO_*f*_, Zr(1.0)BVO_*f*_, and Zr(3.0)BVO_*f*_ films deposited on FTO/glass substrates. LSV curves were obtained in pH 6.86 phosphate buffer solution under photoirradiation of these thin films with a 300 W Xe lamp.

Upon photoirradiation with a Xe lamp (E_g_ > 2.4 eV) in a standard three-electrode system, BiVO_4_ and Zr-doped BiVO_4_ films undergo carrier generations and separations. Positive holes that have accumulated at surfaces of these films oxidize water into O_2_, whereas electrons flow through the outer circuit to generate photocurrents. Hence, the PEC activity for water oxidation can be evaluated by the magnitude of anodic photocurrents during photoirradiation. Figure 7 shows typical current density-voltage scans of BVO_*f*_, Zr(0.5)BVO_*f*_, Zr(1.0)BVO_*f*_, and Zr(3.0)BVO_*f*_ under photoirradiation. None of the films provided appreciable anodic photocurrents when the current density-voltage scan was performed in the dark, indicating that the observed currents were derived from PEC water oxidation. Similar to the above-described results for photocatalytic O_2_ evolution in the powder photocatalytic system, significant improvement of PEC water oxidation was achieved for Zr doping with a relatively low content (Zr(0.5)BVO_*f*_), whereas the films with higher contents of doped Zr became detrimental due to the structural change from active *ms*-BiVO_4_ to inactive (or less active) *ts*-BiVO_4_ and/or *tr*-BiVO_4_ crystals.

## Conclusions

In this study, we proved the effectiveness of Zr doping into an *ms*-BiVO_4_ crystal for improvements of its photocatalytic and PEC functions for water oxidation to produce O_2_. The doped Zr was assumed to be replaced with the Bi site: due to the replacement of electron-rich Zr, *n*-type doping would occur, similar to Mo and/or W doping in the *ms*-BiVO_4_ crystal. However, detailed investigation of the in crystallographic and electric structures, e.g., the actual doping site(s) of Zr in the crystalline lattice of *ms*-BiVO_4_, and quantitative evaluation of carrier densities of samples with and without doped Zr have not been performed. Further studies along these lines are now in progress.

## Author contributions

SI managed all the experiments and wrote the manuscript; TK performed experiments for fabrication of powder and thin film samples, and evaluated photocatalytic and PEC properties of them; YH performed experiments for fabrication of powder samples, and evaluated photocatalytic properties of them; NK performed experiments for fabrication of thin film samples and evaluated PEC properties of them; TH performed experiments for fabrication of powder and thin film samples, evaluated photocatalytic and PEC properties of them, and characterized them by SEM; MR performed experiments and analyses for characterization of powder and thin film samples by XRD and Raman spectroscopy; MI performed experiments and analyses for characterization of powder and thin film samples by XRD; TS performed experiments and analyses for characterization of powder and thin film samples by XRD and Raman spectroscopy, and summarized all the experimental data.

### Conflict of interest statement

The authors declare that the research was conducted in the absence of any commercial or financial relationships that could be construed as a potential conflict of interest.
